# Variability of blue carbon storage in arid evaporitic environment of two coastal Sabkhas or mudflats

**DOI:** 10.1038/s41598-023-39762-7

**Published:** 2023-08-05

**Authors:** Zulfa Ali Al Disi, Khaled Naja, Sankaran Rajendran, Hadil Elsayed, Ivan Strakhov, Hamad Al Saad Al-Kuwari, Fadhil Sadooni, Maria Dittrich, Jassim Abdulla A. Al-Khayat

**Affiliations:** 1 Environnemental Science Center, Qatar University, P.O. Box 2713, Doha, Qatar; 2 Biomedical Research Center, Qatar University, P.O. Box 2713, Doha, Qatar; 3 Biogeochemistry Group, Department of Physical and Environmental Sciences, University of Toronto Scarborough, 1065 Military Trail, Toronto, M1C 1A1 Canada

**Keywords:** Carbon cycle, Biogeochemistry

## Abstract

Coastal Sabkhas are mudflats found in arid coastal regions that are located within the supratidal zone when high rates of evaporation lead to high salinity. While evaporitic minerals often accumulate underneath the surface, the microbial mats are present on the surface of Sabkhas. Coastal Sabkha, an under-studied ecosystem in Qatar, has the potential to store blue carbon. In the present study, we investigated the carbon storage capacity of two Sabkhas from contrasting geological backgrounds. The spatial and temporal variabilities of the carbon stocks were examined. The results showed that both studied Sabkhas exhibit a considerable potential for soil carbon storage with carbon stocks of 109.11 ± 7.07 Mg C ha^−1^ and 67.77 ± 18.10 Mg C ha^−1^ in Dohat Faishakh and Khor al Adaid Sabkha respectively. These values fall within the reported range for carbon stocks in coastal Sabkhas in the region (51–194 Mg C ha^−1^). Interestingly, the carbon stocks in the sediments of the Sabkhas were higher than those in the sediments of Qatari mangroves (50.17 ± 6.27 Mg C ha^−1^). These finding suggest that coastal Sabkhas can serve as blue carbon ecosystems in arid environments.

## Introduction

The term “blue carbon” was coined a decade ago to describe the vital contribution of coastal ecosystems in sequestrating atmospheric carbon dioxide thus having significant carbon stocks and fluxes^1,2^, and to draw attention to the degradation of coastal ecosystems that require urgent conservation and restoration efforts to mitigate the effects of climate change^3,4^.

Sabkha is the Arabic term for broad, flat inter-and supratidal salt flats lacking vascular plants, and is prone to varying frequency and duration of inundation^5,6^. Sabkhas are formed in an arid climate when the rate of evaporation exceeds the rate of rainfall; thus, evaporite deposition prevails either at the surface or within sediments^6^. Ancient Sabkha sequences are important oil and gas reservoirs^7^. Sabkhas has a geographically broad habitat range, with their presence in Southeast Europe, the California coast, Mexico, the Middle East and North Africa region, Australia, and the Arabian Peninsula^8^. Based on their location relative to the shoreline, Sabkhas are categorized into two types: coastal and inland Sabkhas^9^. Coastal Sabkhas are generally found along the shoreline of arid regions. These Sabkhas are continuously fed with saline seawater to replace evaporative losses and contain siliciclastic or carbonate sediments^6,9^. These Sabkhas are typically flooded periodically during spring tides and when northerly winds strongly drive seawater inland^5,10^. Coastal Sabkhas are often characterized by the presence of living microbial mats^11^. These mats are composed of diverse communities of microorganisms, such as cyanobacteria, diatoms, and other types of algae, as well as fungi and bacteria^12^. These microbial mats play an essential role in the ecosystem by providing a source of primary production, stabilizing sediments, and influencing biogeochemical cycling^13^.

Studies on vegetated coastal habitats, such as mangroves, seagrass, and Sabkhas, have established their capacity to sequester and store significant amounts of organic carbon^14,15^. Recent studies suggest that coastal Sabkhas, which are dynamic and productive ecosystems, have the potential for carbon sequestration^8,16^. Thereupon, coastal Sabkhas could represent a unique, but overlooked, blue carbon ecosystem that should be considered when modelling the global carbon budget^17^.

Unlike terrestrial ecosystems, carbon sequestered in coastal sediments can be ample and can remain stored for a long time, resulting in sizable carbon stocks^18^. Coastal ecosystems are approximately two to four times more effective than forests at sequestering carbon dioxide on a per-area basis per year, and they can store up to twice the amount of carbon in their soil and sediment^19^. Despite the importance of these habitats, more than half of blue carbon ecosystems have been lost or degraded within the last 50 years at an alarming rate^20^. Once these habitats have been disturbed, they no longer act as carbon sinks and turn into a source that liberates stored carbon into the atmosphere. Therefore, it is important for the community, including scientists, policymakers, and the public, to study the global carbon cycle and its relationship with coastal blue carbon ecosystems. This knowledge can inform effective strategies for carbon sequestration and the restoration and conservation of these vital habitats. Moreover, there is still much that we do not understand that could explain the variability in carbon storage across blue carbon ecosystems, including coastal Sabkhas.

The Sabkhas of Qatar have been of interest since the 1960s, as they are considered an analog of ancient sedimentary sequences^21^. Previous investigations of coastal Sabkhas in Qatar have either documented their geomorphology^10^, but have mainly focused on dolomite-forming processes and the role of microorganisms^22–24^. While previous studies have primarily focused on the geomorphology and dolomite-forming processes of these Sabkhas, overlooking the carbon storage potential and its dynamics. To bridge this gap, a thorough investigation is needed to quantify the carbon stocks within the sediments of the coastal Sabkhas, while also exploring the biogeochemical factors that contribute to the spatial variations in carbon storage. Additionally, understanding the drivers of seasonal variability in carbon stocks is crucial for comprehending the functioning and resilience of these ecosystems. By addressing these research gaps, valuable insights can be gained to inform global carbon budget modeling and contribute to the effective management and conservation of these vital coastal blue carbon ecosystems.

This study reports the blue carbon potential of Qatar’s coastal Sabkhas. Our objective was to perform a comprehensive analysis of carbon stocks in the Qatari coastal Sabkhas. We quantified the carbon stock in two coastal Sabkhas, analyzed the potential biogeochemical factors affecting the spatial variability of these stocks, and discussed the main drivers of their seasonal variability.

## Material and methods

### Site description

**Figure 1:**
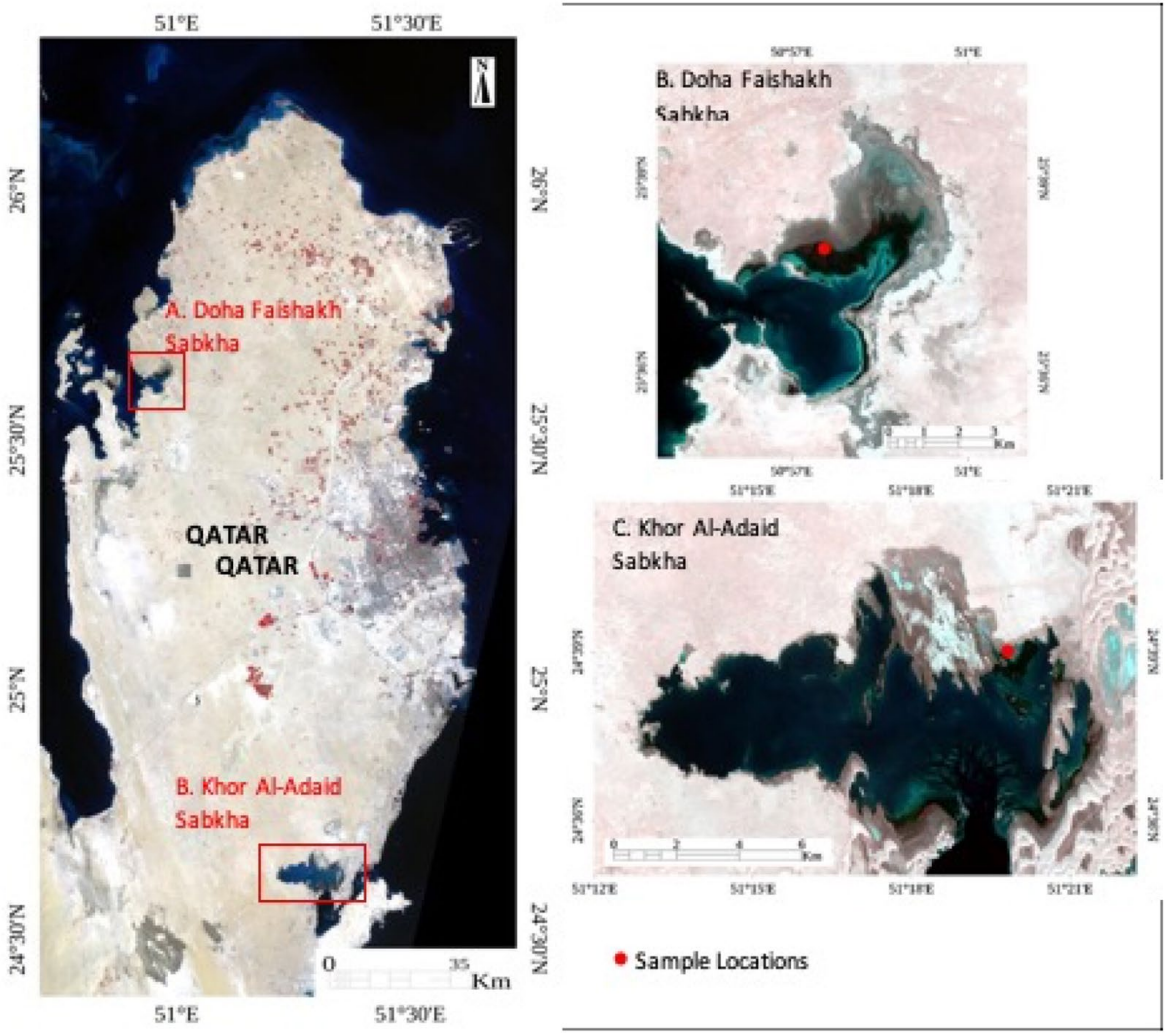
Sentinel-2 satellite image showing Qatar and the sampling regions (**A**). Dohat Faishakh Sabkha (**B**). Khor Al-Adaid Sabkha (**C**).

Two coastal Sabkhas in the state of Qatar were selected for this study (Fig. 1). Khor Al-Adaid (KA) Sabkha is located southeast of Qatar, and Dohat Faishakh (DF) Sabkha is located on the northwest coast of Qatar. Sampling points were selected based on previous studies^22,25^.

**Figure 2:**
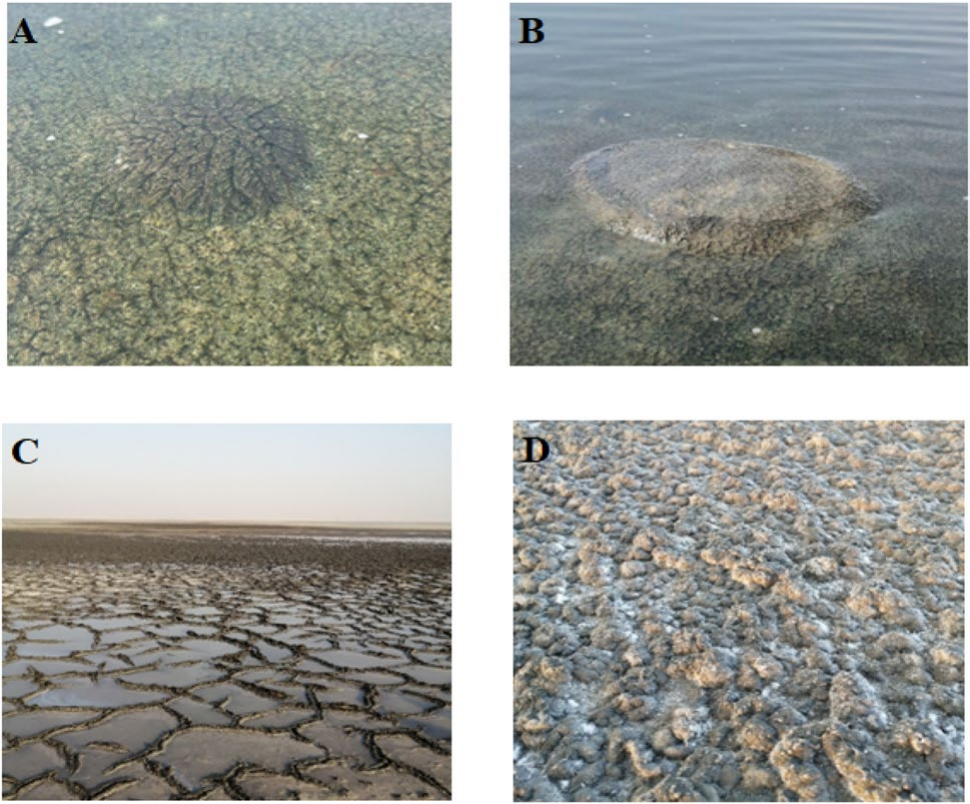
Photographic images of different microbial mats at KA Sabkha (**A** and **B**) and DF Sabkha (**C** and **D**).

The KA Sabkha is a large tidal embayment consisting of two marginal inland lagoons. It is a hypersaline Sabkha covered with microbial mats (Fig. 2), surrounded by large sand dunes; the sediments of this Sabkha are dominated by siliciclastic particles^10^. DF Sabkha is an evaporitic environment covered with microbial mats, and its sediments are dominated by gypsum and carbonate minerals that formed during the Holocene^26^.

### Sample collection and preparation

Core samples were collected during different seasons during the period 2021–2022 (Table 1). Three sediment cores were collected from each sampling point using a 6 cm diameter plastic core. Sediment cores were sectioned into 5 cm layers, starting from the surface layer down to a depth of 35 cm (refusal depth). Five grams of each sectioned layer were transferred to sterile tubes and freeze-dried. The freeze-dried sediments were manually ground using a mortar and pestle before geochemical analysis. Prior to the analysis, large visible crystals of gypsum were removed from the cores collected from the DF Sabkha.

**Table 1:** Dates and locations of sampling points from DF and KA Sabkhas.

Sabkha	Collection date	GPS coordinates	Salinity (‰)
DF	15-Dec-21	25° 38′ 8.17″ N 50° 57′ 36.48″E	261
	22-Feb-22	25° 38′ 4.74″ N 50° 57′ 35.532″ E	140
	25-Oct-22	25° 38′ 4.74″ N 50° 57′ 35.532″ E	310
KA	20-Dec-21	24° 39′ 8.712″ N 51° 20′ 9.732″ E	79
	17-Feb-22	24° 38′ 45.870″ N 51° 19′ 35.760″ E	47
	24-Oct-22	24° 38′ 17.5″ N 51° 18 ′24.4″ E	89

The bulk density (g cm^−3^) was determined using the core method^27^. The core was collected in a way that does not cause compaction, carefully sectioned without losing material, and then dried at 105 °C for 2 d. Bulk density was measured by dividing the oven-dried soil sample by the internal volume of the cylinder.

### Major and trace elements analyses

For the analysis of major and trace elements (Ca, Na, Be, Mg, Al, P, K, Sr, Mn, Fe, V, Cr, Co, Ni, Zn, As, and Mo), sediment samples were digested as follows:1 mL of 50% HNO_3_ and 3 mL of HF were used to digest 100 mg of each sediment sample in a tightly closed polytetrafluoroethylene container maintained on a hot plate at 160 °C for 48 h. After evaporation to dryness, 1 mL of 55% HClO_4_ was added to the container, which was heated at 160 °C until the acid evaporated completely. As soon as the sample was cooled to 25 °C (room temperature), 50% HNO_3_ was added, and the sample was heated for 12 h at 160 °C. Subsequently, the solution was cooled to room temperature and diluted with 10% HNO_3_^28^. The elemental composition was determined by inductively coupled plasma mass spectrometry (ICP-MS) using a PerkinElmer Optima 5300 DV instrument.

### Mineralogical composition

The bulk mineralogical composition of the sediments was determined using a PANalytical multipurpose Empyrean X-ray diffractometer. Analysis of the XRD spectra was performed using the Crystal Impact Match software, version 3.15. The amounts of minerals in each mixture were semi-quantitatively estimated using the Match software.

### Carbon stock measurements

The sediment samples were processed to determine their total carbon (TC) content using a CHNS Skalar Primacs SNC-100 TN/TC/IC analyzer. First, the sediment samples were ground to a particle size of approximately 0.05 mm, and then 75 to 125 mg of the sediment was used for the analysis. To determine the TC content, the samples were combusted with pure O_2_ at 1200 °C to enable complete oxidation of carbon to CO_2_. Subsequently, the CO_2_ produced was measured using infrared spectroscopy (IR). For the analysis of inorganic carbon (TIC), the samples were treated with phosphoric acid to produce CO_2_, which was detected by IR. The organic carbon content was determined by subtracting TIC from TC.

To calculate the sediment organic carbon stocks (C_org_) in each layer, the following formula was used:

$${\text{Sediment}}\;{\text{carbon}}\;{\text{(Mg}}\;{\text{C}}\;{\text{ha}}^{ - 1} {)} = {\text{Bulk}}\;{\text{density}}\;({\text{g cm}}^{ - 3} ) \times {\text{Depth}}\;{\text{increment}}^{29} \times \% {\text{TOC}}$$

C_org_ at a specific depth was estimated as the sum of the C_org_ stocks in all sediment layers. We extrapolated the sediment C_org_ stocks per unit area to a depth of 1 m to enable a comparison with the results reported in previous studies.

### Satellite data and image processing

Multispectral Imager (MSI) of Sentinel-2 has 13 bands in the VNIR to SWIR spectral region with spatial resolutions of 10, 20, and 60 m^30^. In this study, we obtained cloud-free MSI Level-1C data from the European Space Agency's Copernicus Open Access Hub that was closest in time to the date of our field sampling. Table 2 provides details on satellite data acquisition, including the date, cloud cover percentage, and the use of both Sentinel-2A and Sentinel-2B for the Dohat Faishakh Sabkha and Khor Al-Adaid Sabkha, with specific information on their launch dates, orbits, equatorial crossing times, field of view, and repeat cycles. The data were preprocessed using the Sentinel Application Platform (SNAP) program, which includes the Sen2Cor plugin and Sentinel-2 Toolbox (http://step.esa.int/main/toolboxes/snap/)^31,32^. In this study, the soil salinity of the Sabkhas was image processed using the spectral bands of MSI and indices, namely the Normalized Difference Salinity Index (NDSI) (band11-band12)/(band11 + band12), which was used for inland Sabkha of Qatar by^33^ and available in the Index database (IDB) for Sentinel-2 remote sensing indicators (https://custom-scripts.sentinel-hub.com/custom-scripts/sentinel-2/indexdb/).

**Table 2:** List of Sentinel-2 data that used in the study.

Sl. no.	Date	Data	Cloud cover %
Dohat Faishakh Sabkha			
1.	05.12.2021	S2A_MSIL1C_20211205T071251_N0301_R106_T39RVJ_20211205T080841	0.0
2.	23.02.2022	S2A_MSIL1C_20220223T070901_N0400_R106_T39RVJ_20220223T092332	0.0
3.	26.10.2022	S2B_MSIL1C_20221026T070959_N0400_R106_T39RVJ_20221026T075522	0.013
Khor Al-Adaid Sabkha			
4.	20.12.2021	S2B_MSIL1C_20211220T071309_N0301_R106_T39RWH_20211220T081300	12.8586
5.	18.02.2022	S2B_MSIL1C_20220218T070929_N0400_R106_T39RWH_20220218T090947	0.0
6.	26.10.2022	S2B_MSIL1C_20221026T070959_N0400_R106_T39RWH_20221026T075522	0.032

### Statistical analysis

The statistical analysis package IBM SPSS Statistics, Version 28.0.1.0 (142), was used to perform all statistical analyses in this study. All results are expressed as the mean ± standard deviation. Data were tested for normality and homogeneity of variance to ensure that they satisfied the assumptions of parametric methods. Independent t-tests and one-way analysis of variance (ANOVA) were used to assess differences among the sites in terms of sediment characteristics. The Pearson's correlation coefficient was computed to test the possible correlation between organic carbon content and depth.

## Results

### Downcore profiles of major and minor elements

**Figure 3:**
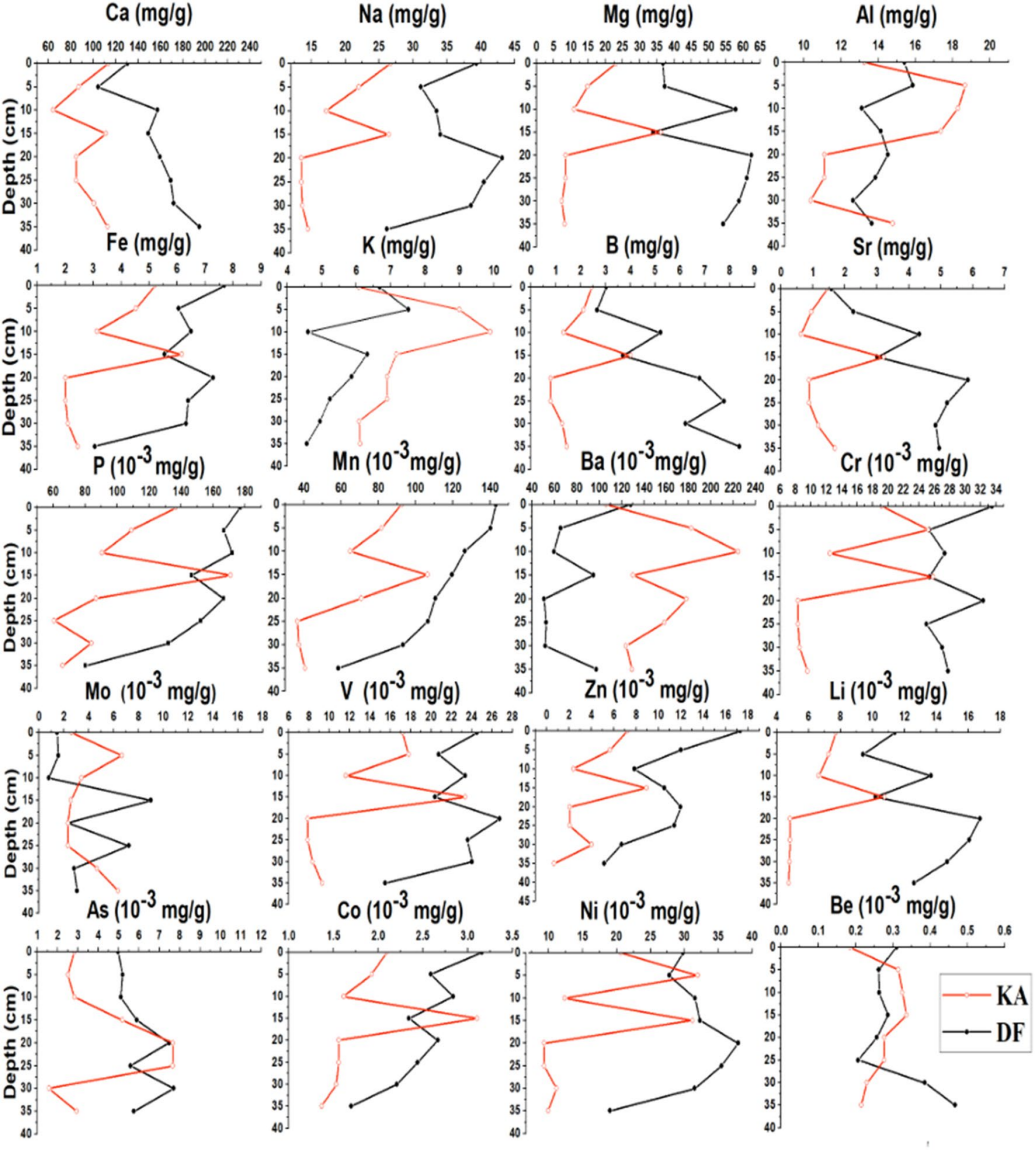
Depth profiles for average concentrations (mg/g) of major elements and trace elements sampled from sediments of KA (red lines) and DF (Black lines).

The chemical characteristics of the major elements in the sediment samples collected during different seasons from the DF and KA Sabkhas are shown in (Fig. 3).

To account for potential variation, average concentrations of major and minor elements were calculated across different layers and seasons. The sediments from DF Sabkha showed significantly higher concentrations of major and minor elements than those from KA Sabkha (P = 0.008). For instance, the sediments from KA contained an average of 64–114 mg/g calcium across different layers, whereas the sediments from DF had an average of 95–231 mg/g calcium. Similarly, the average Na concentration was 14–27 mg/g in KA and 16–43 mg/g in DF. However, the average concentration of K was higher in KA than in DF, with 6–10 mg/g and 5–8 mg/g, respectively (P = 0.007). The Ba concentrations were also higher in KA than in DF, with sediments from KA having significantly higher concentrations (P = 0.002) of Ba (0.12–0.22 mg/g) compared to DF sediments (0.03–0.1 mg/g).

**Figure 4:**
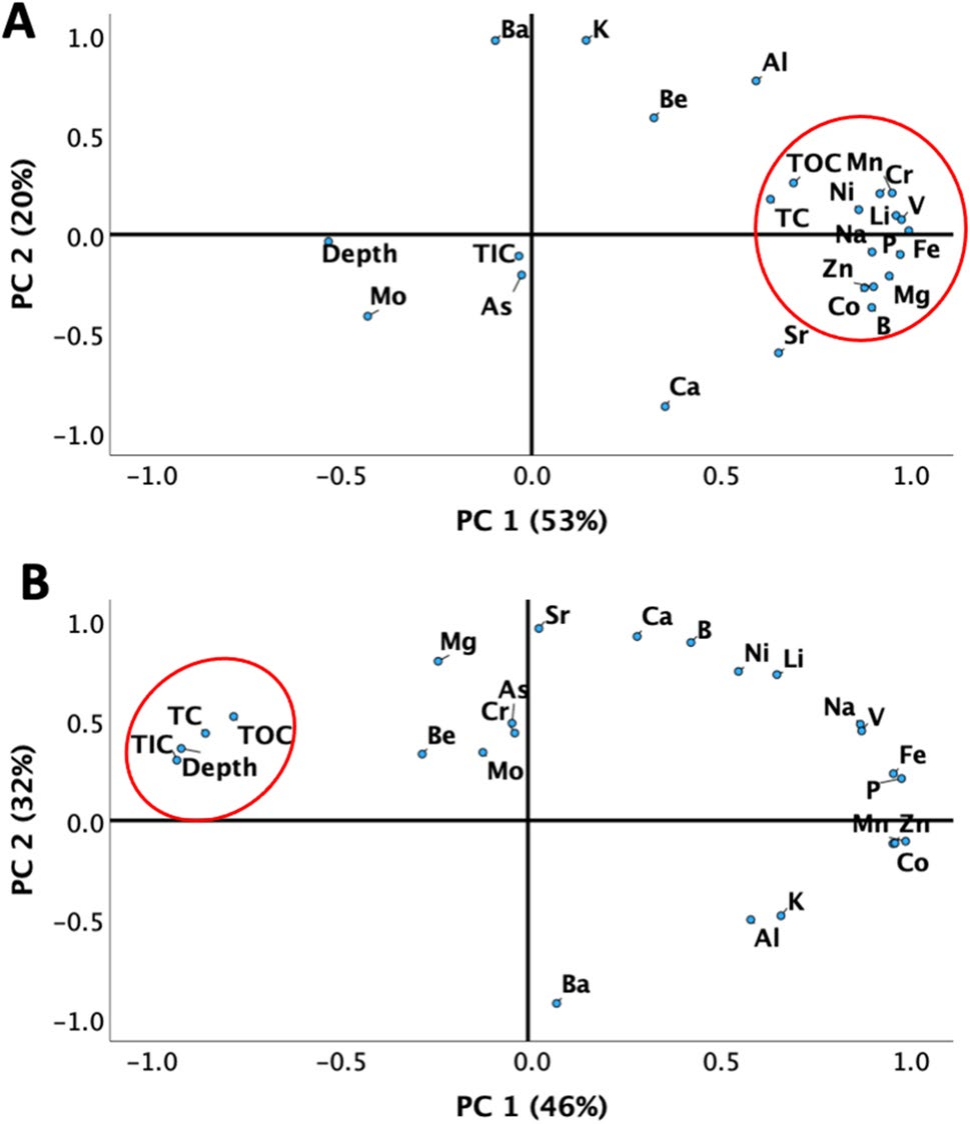
PCA results showing the relationship between elements, depth, and total organic content in: (**A**) KA and (**B**) DF Sabkhas. Distinct patterns are observed between the two locations.

Principal Component Analysis (PCA) was employed to gain further insights into the relationships between the studied variables and TOC. The PCA results are presented in (Fig. 4), which shows the relationships between the major & minor elements, depth, and total organic content in KA and DF Sabkhas. The first two principal components (PC1 and PC2) were found to explain 73% and 78% of the variability in the data of KA and DF, respectively, indicating their significant contribution to the analysis. The PCA results show that there is a strong correlation between TOC and depth in DF. However, in KA, TOC appears to have no relationship with depth, and is more influenced by elemental concentrations.

### Mineralogical composition

**Figure 5:**
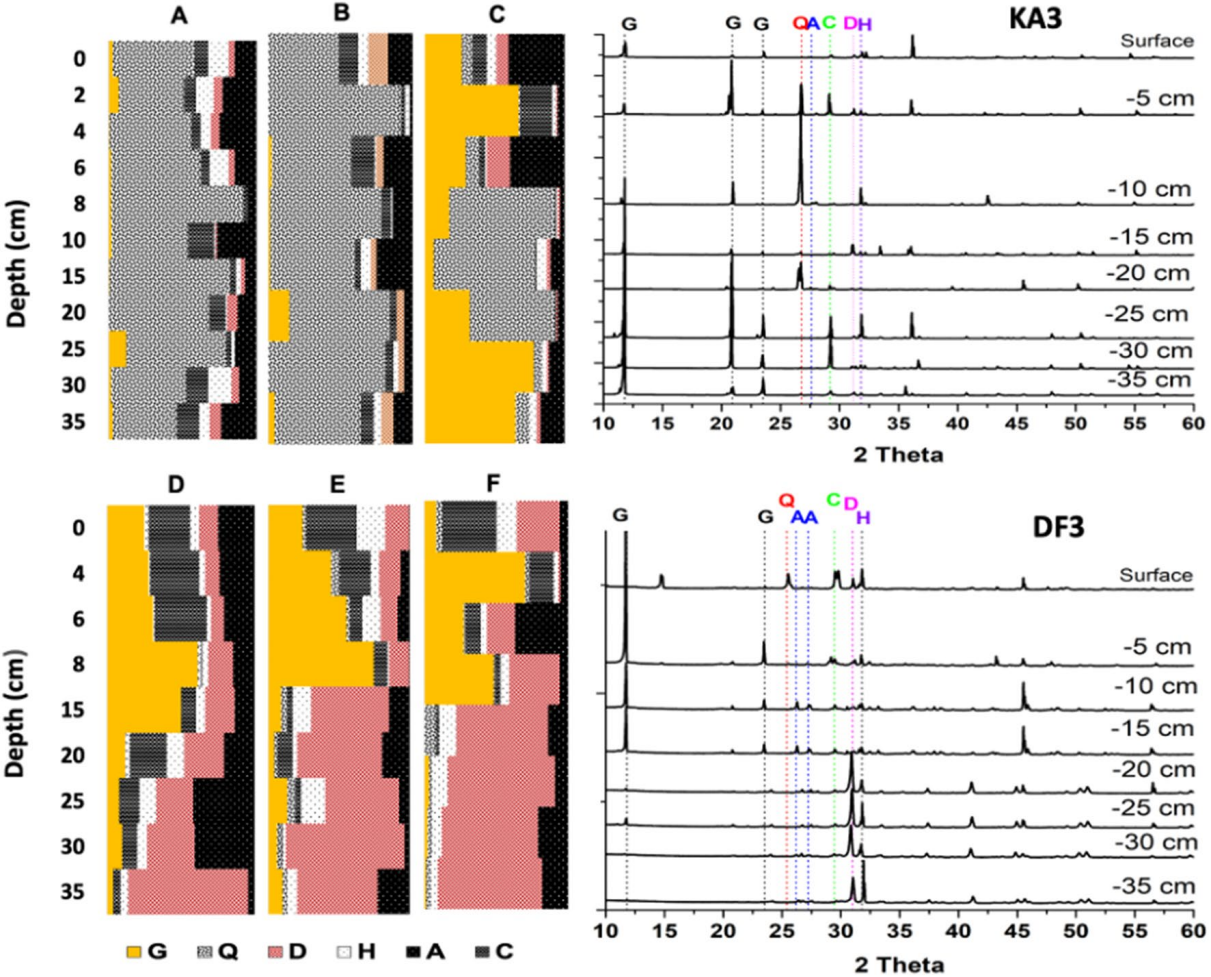
Mineralogy depth patterns semi-quantitatively calculated using XRD data of the core sediment samples collected from DF and KA Sabkha in different seasons: (**A**) KA1-Dec 21, (**B**) KA2, Feb 22, (**C**) KA3, Oct 22, (**D**) DF1, Dec 21, (**E**) DF2, Feb 22, and (**F**) DF3, Oct 22. Examples showing XRD patterns of KA3 and DF3 are illustrated in the right panel. Q: Quartz, C: Calcite, D: Dolomite, A: Aragonite, G: Gypsum, H: Halite.

The XRD spectra analysis of sediments collected from the two Sabkhas showed a consistent mineral profile throughout the different seasons, except for those collected from KA, which displayed relatively higher variability (Fig. 5). Interestingly, the depth profiles revealed significant differences in mineral abundance. For instance, quartz was abundant in different layers of KA sediments, with increasing amounts of gypsum observed in the sediments collected in October 2022. In contrast, sediments collected from DF contained gypsum, calcite, and dolomite. Notably, the abundance of dolomite increased in the deeper layers of the sediment cores.

### Sediments organic carbon and carbon stocks

**Figure 6:**
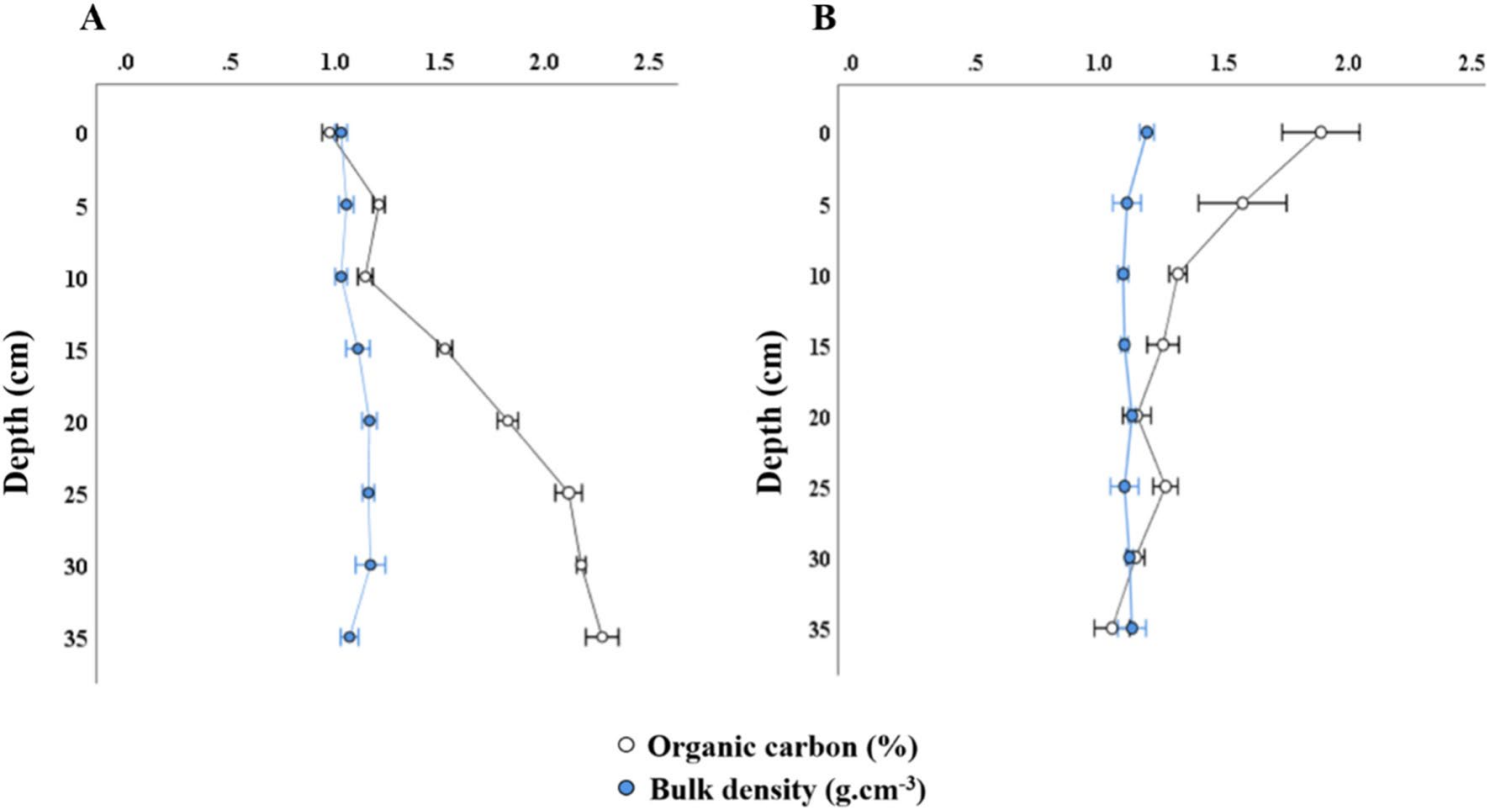
Percentage of organic carbon and bulk density variations along with depth for each coastal Sabkha. **A**) DF and **B**) KA. Results are represented as mean ± SD.

The sediment organic carbon followed a different depth trend in each Sabkha (Fig. 6). Pearson's correlation was calculated to test the potential relationship between organic carbon content and depth in each Sabkha.

In KA Sabkha, a significant negative correlation between organic carbon content and depth (r =  − 0.85, P < 0.01). Conversely, in DF Sabkha, as strong and positive correlation was identified between organic carbon content and depth (r = 0.97, P < 0.01). Interestingly, no significant difference in the bulk densities of the sediments between the two Sabkhas. Additionally, no significant correlation was observed between the bulk density and organic carbon in each of the two studied Sabkhas.

**Figure 7:**
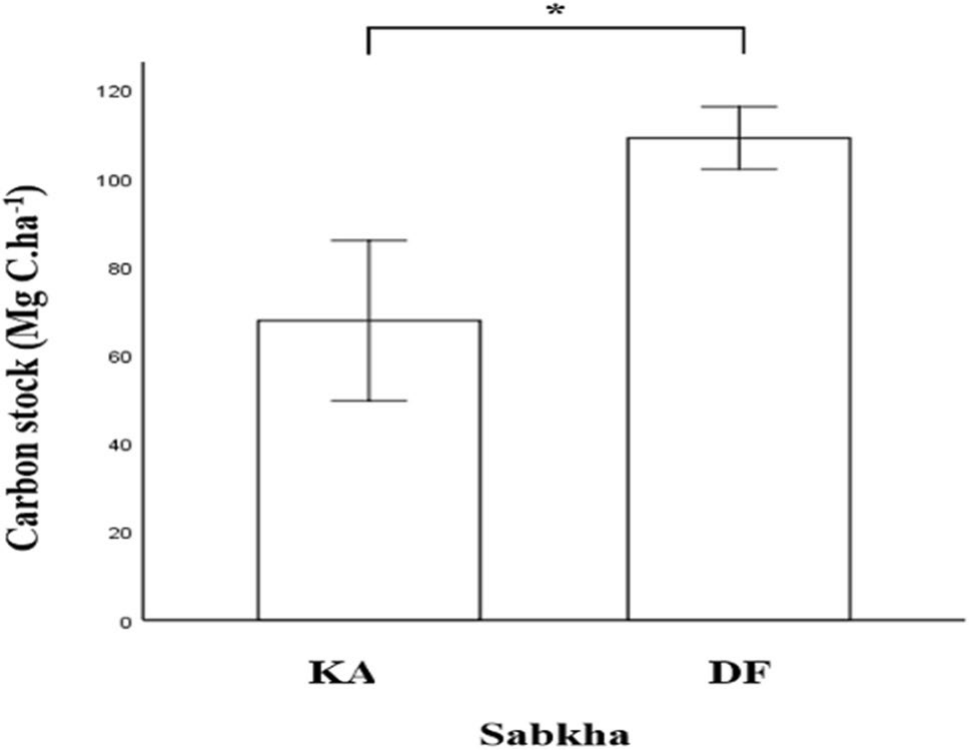
Carbon stock in the two studied Sabkhas. Results are represented as mean ± SD. *P < 0.05, independent sample t-test.

To estimate the sediment carbon stocks in each Sabkha, the mean sediment carbon stocks across seasons were calculated (Fig. 7). Remarkably, the carbon stocks, averaged for all seasons, in DF (109.1 ± 7.1 Mg C ha^−1^) were significantly higher (P = 0.021) than in KA (67.8 ± 18.1 Mg C ha^−1^).

### Seasonal carbon stocks

**Figure 8:**
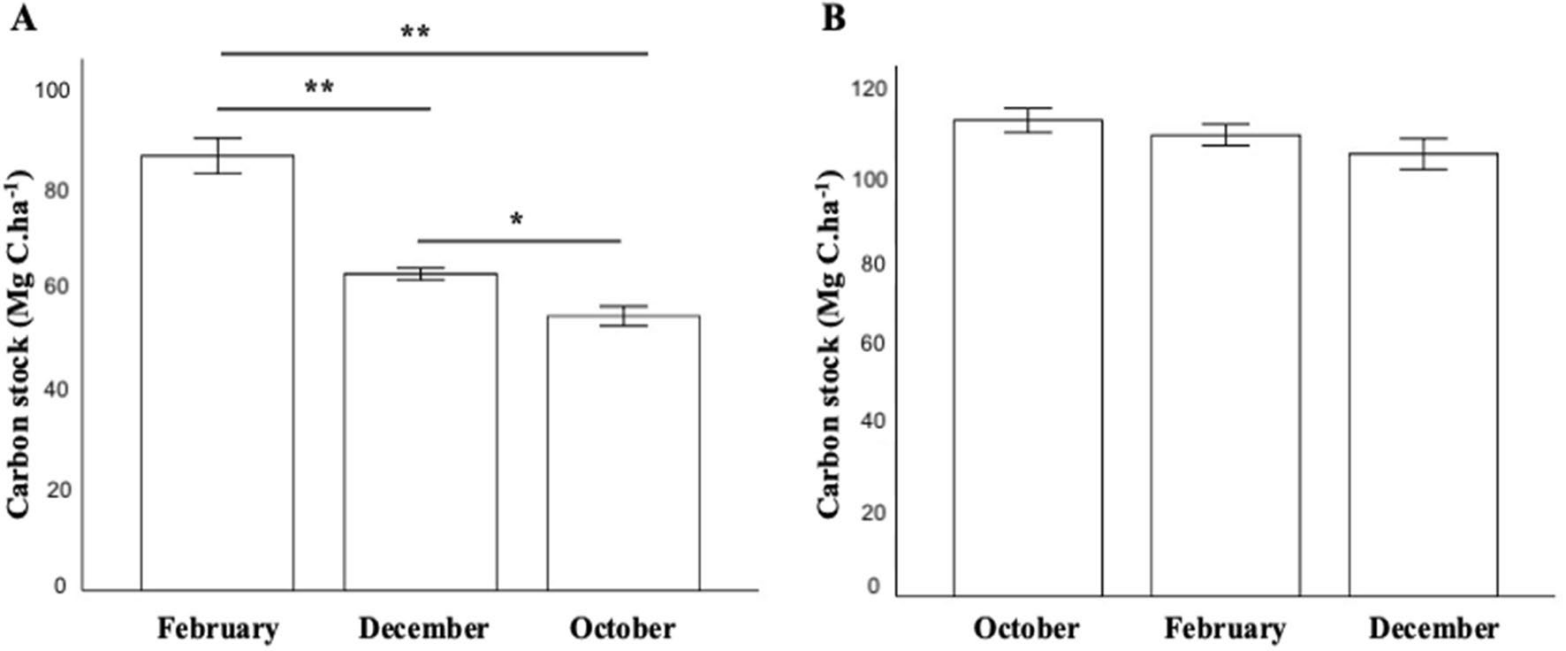
Carbon stock in each Sabkha in different seasons. (**A**) KA Sabkha and (**B**) DF Sabkha. Results are represented as mean ± SD. *P < 0.05, **P < 0.001, one-way ANOVA.

Carbon stocks were calculated for each Sabkha in different seasons. The results are shown in (Fig. 8). Significant variations in carbon stocks within KA across different seasons were observed, while in DF, these variations were relatively minor.

### Salinity mapping using remote sensing

**Figure 9:**
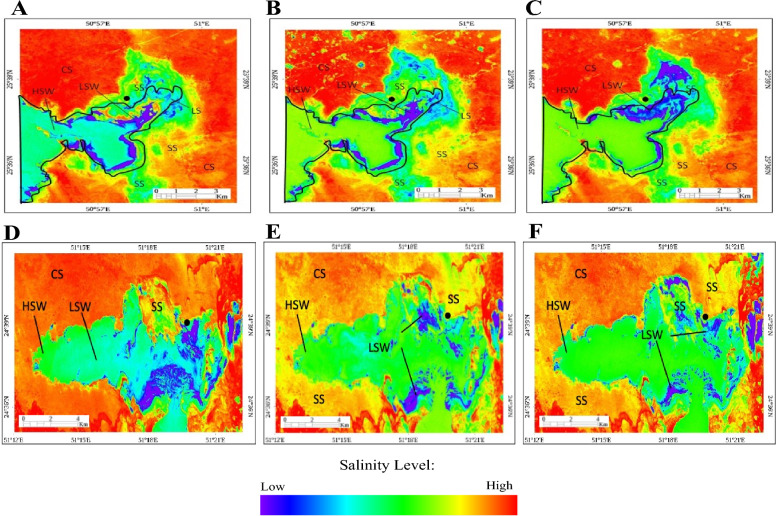
Normalized Difference Salinity Index (NDSI) images of MSI of the DF Sabkha acquired on (**A**) December 5, 2021, (**B**) February 23, 2022, and (**C**) October 26, 2022, showing the area of saline soil (SS); Carbonate soil (CS); High Saline Water (HSW) and Low Saline Water (LSW). Normalized Difference Salinity Index (NDSI) images of MSI of the KA Sabkha acquired on (**C**) December 20, 2021, (**D**) February 18, 2022, and (**F**) October 26, 2022, showing the area of saline soil (SS); Carbonate soil (CS); High Saline Water (HSW) and Low Saline Water (LSW)). • locates sample site.

The NDSI images obtained for dates closet to the field sampling period are illustrated in Fig. 9). These images allow for visualizing the spatial distribution of soil salinity around and within the studied Sabkhas. The maximum salinity of the soil is interpreted in red around the Sabkha because of the high reflectance of the saline soil (carbonate soil, CS) of the area. The images show salt-crusted saline soil (SS), which contains gypsum, halite, and anhydrite that occur within the Sabkha and around the Sabkha appear yellow to cyan. All images demonstrate a gradual increase in salinity over time in the soil of the Sabkha, potentially due to changes in the arid climate^33^. Moreover, the changes in salinity is influenced by the prevailing hydrodynamics in the Arabian Gulf^34^.

## Discussion

One of the understudied coastal ecosystems is Sabkha, which has the potential to store organic carbon. The coastal Sabkhas extend along the shoreline of Qatar. The carbon stocks in the studied coastal Sabkhas were entirely in the soil pools. Our data showed that both Sabkhas have a high soil carbon storage potential (67.8 ± 18.10 and 109.11 ± 7.07 Mg C ha^−1^). The carbon stocks observed in both Sabkhas align with the range of carbon stocks documented in previous studies conducted on coastal Sabkhas and similar coastal environments. To provide a broader perspective, Table 3 presents a summary of carbon stocks measured in different countries across various coastal environments.

**Table 3:** List of comparative recent studies of carbon stocks in different coastal environments.

Country	Setting	C_org_ stock, Mg C ha^−1^	References
UAE	Coastal Sabkhas	51–120	^5^
Saudi Arabia	Coastal Sabkhas	63–194	^8^
Qatar	Coastal Sabkhas	68–109	This study
Netherlands	Mudflats	62–179	^35^
United Kingdom	Salt Marshes	43–94	^29^

Interestingly, the two studied coastal Sabkhas showed higher carbon stocks than the soil stocks of Qatari mangroves (50.17 ± 6.27 Mg C ha^−1^)^36^. This is consistent with other studies that reported the low capacity of mangrove sediments to act as carbon sinks^37,38^. This shows that mangroves have a limited capacity for soil carbon storage when compared to coastal Sabkhas in low-rainfall, hypersaline areas and indicates that coastal Sabkhas could be considered substantial blue carbon ecosystems in arid environments.

Our results show that sediment organic carbon followed a distinct depth trend in each Sabkha. The organic carbon in DF increased with depth, which could be explained by the organic carbon-mineral interaction, since the top layers are dominated by gypsum, whereas the deep layers are dominated by dolomite. In contrast, the organic carbon in KA decreased with depth, which can be attributed to the allochthonous deposition of carbon on the topsoil. This difference underscores the variability in organic carbon stabilization among coastal Sabkhas, which may be influenced by a range of factors such as microbial community composition, mineralogy, and hydrology^39^.

Different biogeochemical backgrounds may affect the retention of carbon stocks in sediments, and the interactions that govern organic matter preservation are complex^40^. Published studies agree on the high variability of organic carbon stocks, which can occur within relatively small areas^41^. Our emerging data show that DF Sabkha exhibits significantly higher carbon stocks than KA Sabkha. Based on field measurements and mapping satellite data (Table 1 and Fig. 9), salinity may be the primary explanatory factor. The salinity levels in DF Sabkha, ranging from 140 to 310, are significantly higher compared to those in KA Sabkha, which range from 47 to 89 ‰**.** The effect of salinity on carbon storage can be attributed to the fact that high salinity can reduce the activity of most microbes or create an environment that is inhospitable to many microorganisms^42,43^, thus reducing organic carbon output. Moreover, high soil salinization can normally reduce the emissions of sediment organic carbon because the stiff saline layer largely restricts its redox reactions^44,45^. This is consistent with many studies showing a positive relationship between soil salinity and organic carbon^46–48^. However, some studies have reported a negative impact of high salinity levels on the preservation of organic matter^49^. Nevertheless, the relationship between organic carbon preservation and salinity in Sabkhas is complex and can be influenced by several other factors.

Another factor that could explain this spatial variability in carbon stocks is the mineralogy. Organic matter-mineral interactions are essential components of the global carbon cycle and contribute to the preservation of organic matter^50,51^.

As expected, our XRD and mineralogy patterns for bulk sediment obtained from the two studied Sabkhas showed that KA, a siliciclastic carbonate Sabkha, has a more diverse range of mineral types than DF, a pure carbonate Sabkha, which could cause greater heterogeneity and ultimately reduce the capacity of organic carbon storage of the Sabkha^52^. These results are in line with a study that suggested that the petrophysical characteristics of a siliciclastic-carbonate reservoir are more complex than those of pure carbonate or siliciclastic reservoirs, which might reduce the quality of the reservoir^52^.

The concentration of major elements may have a significant influence on the preservation of carbon, although the impact of these elements on organic matter preservation in Sabkha environments is complex. For instance, calcium has been shown to play a role in the preservation of organic matter^53^ by forming calcium-organic complexes that are resistant to degradation^54^. Our results showed significantly higher calcium concentrations in DF sediments than in the KA sediments. Furthermore, the concentration of trace metals can serve as an indicator of redox conditions in sediments^55^. Our data showed average higher concentrations of iron^56^ and molybdenum (Mo) in the sediments of DF, which is consistent with previous research indicating that trace elements such as iron, manganese, and molybdenum that are present in the Sabkha sediments can facilitate the preservation of organic matter^57^.

The PCA results indicate that there is considerable difference in the association between TOC, TIC, elements, and depth in the two studied Sabkhas. This difference suggests that the factors affecting the distribution of organic matter in the two locations may be distinct, requiring further investigation into the biogeochemical mechanisms that control the preservation of organic matter in Sabkhas.

Previous studies have reported seasonal variations in the biogeochemical characteristics of Sabkha^58,59^. In this study, the seasonal variability of carbon stocks in each Sabkha was investigated. The carbon stocks in KA Sabkha exhibited highly significant variability, indicating that the carbon content in KA is unstable on a seasonal scale and, therefore, likely not a long-term carbon sink.

XRD spectra confirmed that KA Sabkha exhibits considerable variability in mineralogy profiles, which is consistent with the strong seasonal biogeochemical fluctuations observed in this environment. Satellite data also confirmed strong variations in salinity in KA Sabkha. Interestingly, our study found that carbon stocks in KA increased with seasonal salinity, suggesting a potential link between salinity and carbon sequestration in this environment. In contrast, DF Sabkha showed very little seasonal variability in carbon stocks despite variations in salinity. This could be explained by the fact that DF is a stable Sabkha that is not prone to the same level of fluctuations as KA. Overall, our findings highlight the importance of considering both biogeochemical and environmental factors when assessing the potential of Sabkhas as long-term carbon sinks.

## Conclusion

This study provides valuable insights that are directly relevant to coastal Sabkhas in Qatar and the Arabian Gulf, enabling us to compile a more comprehensive inventory of the carbon storage potential of these ecosystems. Our findings demonstrate that coastal Sabkhas have the potential to store significant amounts of organic carbon, ranging from 68 to 109 Mg C ha^−1^. These value are comparable to similar to those of similar coastal ecosystems, which range from 50 to 190 Mg C ha^−1^.

Moreover, our investigations revealed a notable divergence in the organic carbon stocks of the two coastal Sabkhas. The observed differences in carbon stocks may be attributed to variances in their respective biogeochemical characteristics and salinity levels. The KA Sabkha exhibited greater seasonal fluctuations in carbon stocks, while the more stable carbonate-rich DF Sabkha appeared to function as a long-term carbon sink. These findings emphasize the importance of further research including comparative studies with other coastal ecosystems, assessments of carbon fluxes, investigations into climate change impacts, studies of microbial communities, exploration of restoration techniques, and integration of findings into policy and conservation efforts. Conducting such research is crucial for accurately gauging the carbon sequestration potential of these ecosystems and recognizing their critical role in mitigating climate change. Most importantly, this study highlights the urgency of preserving these unique coastal habitats, which have already experienced substantial destruction due to urban development.

### Supplementary Information

Supplementary Information.

## Data Availability

The datasets used and/or analysed during the current study available from the corresponding author on request to any qualified researcher.
